# Comparative Study of the Flipped Classroom and Traditional Lecture Methods in Anatomy Teaching

**DOI:** 10.7759/cureus.64378

**Published:** 2024-07-11

**Authors:** Shweta Jha, Ruchira Sethi, Mukesh Kumar, Gitanjali Khorwal

**Affiliations:** 1 Department of Anatomy, Netaji Subhas Medical College and Hospital, Patna, IND; 2 Department of Anatomy, UNS Autonomous State Medical College, Jaunpur, IND; 3 Department of Microbiology, Netaji Subhas Medical College and Hospital, Patna, IND; 4 Department of Anatomy, All India Institute of Medical Sciences, Rishikesh, IND

**Keywords:** competency based medical education, teaching learning method, didactic lecture, anatomy, flipped classroom

## Abstract

Background

The National Medical Commission (NMC), the regulatory body for medical education in India introduced competency-based medical education (CBME) in 2019. It is an outcome-based learner-centric approach. To implement it, active and innovative learning strategies are being introduced. A flipped classroom (FC) is one such teaching method in which learners are provided learning materials before class with active discussion during teaching sessions. This promotes critical thinking, better retention, and future clinical applicability. This study aimed to compare FC and traditional teaching methods for first-phase medical learners for the anatomy curriculum. The objectives of this study were to evaluate the effectiveness of FC viz-a viz traditional lecture method for certain topics of clinical relevance from the anatomy curriculum and assess students' perception of the FC method.

Methodology

The study was conducted on 96 phase-one medical learners after obtaining approval from the Institutional Ethics Committee. After obtaining informed consent, a simple random sampling method was used to group the students into two groups. For the same topic, one group was taught with the FC method, while the other group was taught using the didactic lecture (DL) method. A crossover was done for another topic. Students were assessed by pre- and post-tests. Students' perceptions' were recorded by a pre-validated questionnaire quantified on a Likert scale.

Results

The difference in posttest scores for the topics taught between the two groups was found to be statistically significant. Perception regarding various aspects of the FC method was affirmative.

Conclusions

The results of this study indicated that FC is advantageous for students. It improves learner engagement and performance, and students' perception of the method was positive. Effective execution of this method requires detailed planning, constant motivation, and consistent efforts. Therefore, this method should be used more often for the benefit of students.

## Introduction

Anatomy is one of the most important basic sciences in the first phase MBBS curriculum, which forms the foundation of clinical education [1]. It is a vast subject as it forms a major part of first-phase undergraduate study hours divided into subsections like gross anatomy, osteology, neuroanatomy, histology, etc. It has been conventionally taught with traditional teaching methods like didactic lectures (DLs), practicals, demonstrations, etc. Anatomy for learners is extremely challenging [2].

The National Medical Commission (erstwhile Medical Council of India) has introduced competency-based medical (CBME) which focuses on a learner-centric approach and attaining clinical competence [3]. To achieve this, higher levels of cognition like synthesis, interpretation, and correlation are required. The advantages of DLs are that they are resource-friendly and can convey information to a sizeable audience, but the pitfall is that they do not inculcate knowledge application and problem-solving skills, which are prerequisites for clinical practice [4].

In 2012, Jonathan Bergmann and Aaron Sams coined the term “flipped classroom.” In this innovative teaching-learning method, events that have been traditionally occurring inside the classroom are held outside and vice versa. It requires giving educational resources like voice-over PowerPoint, tutorials, and lectures in the form of videos, podcasts, notes, and animations for students to use before the class so that class time is used for multiple other activities like case-based discussions, buzz sessions, small group discussions, etc. [5,6]. 

Innovative teaching-learning methods like the flipped classroom (FC) approach can instill clinical relevance in the subject, which will help in critical thinking, interest, and logical reasoning. It is believed that active learning is encouraged by this innovative approach in contrast to DLs, which promote passive learning [7,8]. Enhanced self-directed learning skills, more effective student-teacher interactions, and improved learner engagement and retention are other suggested benefits [9].

Previous literature showed that subjects found FCs better in terms of qualitative data [10-12]. Also, there is a lack of evidence of the effectiveness of FCs on anatomy topics hence the need for this study. The objectives of this study were to assess the effectiveness of the FC as compared to the traditional lecture method in teaching anatomy competencies using multiple-choice questions (MCQs) as an assessment method and to find out students' perceptions of the FC method. 

This article was previously presented as a conference abstract at the 12th International Conference of the Society of Clinical Anatomists, April 4-6, 2024.

## Materials and methods

The present prospective interventional cross-over study was conducted on first-phase undergraduate students to compare the effectiveness of FCs and traditional DL teaching-learning methods. The study was conducted at Netaji Subhas Medical College (NSMCH), Bihta, Patna. The duration of the study was six months.

A total of 96 students were included in the study after obtaining ethical clearance from the Institutional Ethics Committee, Netaji Subhas Medical College (vide letter CREC/2023/42).

Inclusion criteria

All students enrolled in first-year MBBS of NSMCH.

Exclusion criteria

Non-consenting or absent students were excluded from the study.

Out of 100 students enrolled in the first year, four were excluded due to absence. After obtaining informed consent, the participants were randomly divided into two groups, A and B, with 48 individuals in each group. Sensitization of students and faculty was done.

Two topics of clinical relevance were selected for the study. During the first phase, students in group A were taught by the FC method and group B by the DL method. Topic 1 (AN 20.5 - Anatomical basis of varicose veins and deep venous thrombosis) was taught to both groups. Pre- and post-tests were given to both groups before and after the sessions, respectively. These had 20 multiple-choice questions with a weightage of 2 marks each. Scores were recorded.

In the second phase, the two groups were interchanged. Students in group A were taught by DL and those in group B by FC. Topic 2 (AN 18.4 - Ligaments of the knee joint and its applied aspect) was taken up this time. Pre- and post-tests based on the taught topic were given to students, and an assessment was done.

For the FC group, a voice-over PowerPoint was provided one week before the teaching session. A WhatsApp group was created with students and faculty. Additional resources in the form of lecture notes and reading materials were shared with the group. This group was also used to motivate students to study the materials given to them before they attended the teaching session. During the teaching session, students were divided into buzz groups, each of six to seven students. Case scenarios were given by the faculty followed by discussion among the respective groups. This was followed by a conclusion articulated by one representative from each group. Faculty acted as facilitators during these interactive sessions.

For the DL group, no materials were shared before the teaching session. The session was conducted traditionally.

At the end of both phases, a pre-validated questionnaire was used to record the perception of students toward the FC method. The questionnaire was based on a 5-point Likert scale (Strongly Agree, Agree, Neutral, Disagree, and Strongly Disagree). Cronbach's alpha (>0.75) was used to assess internal consistency.

Statistical analysis

A set of 20 multiple-choice questions, each worth 2 marks, was given to students in both groups as a pre-test and post-test assessment tool. SPSS version 20 software was used to analyze data (IBM Corp., Armonk, NY). For descriptive analysis, mean and standard deviation (SD) were used. Categorical variables were analyzed by unpaired t-test. *P*-value < 0.05 was considered significant.

## Results

The quantifiable mean score for each group of learners for the two competency topics is represented in Table 1. The results of the pre-test score for each competency were observed to be statistically nonsignificant, suggesting that the two groups are comparable with the same level of understanding and conceptualization. On the other hand, the post-test scores show a statistically significant difference between the two groups, with higher mean scores for the FC group (*P *< 0.05) (Table 1).

**Table 1:** Mean score of the assessment for the two study groups. SD, standard deviation; S, significant; NS, nonsignificant

Competency no.	Assessment score	Flipped classroom (*n* = 48) (mean score ± SD)	Didactic lecture (*n* = 48) (mean score ± SD)	*P*-value
AN 20.5	Pre-test score	19 ± 5.64	17.5 ± 4.76	0.16 (NS)
	Post-test score	25.12 ± 4.93	19.83 ± 5.57	<0.001 (S)
AN 18.4	Post-test	18 ± 6.13	16.29 ± 3.89	0.10 (NS)
	Post-test score	25.7 ± 3.95	20.29 ± 4.64	<0.001 (S)

Similar results were obtained for topic 2 as well (Table 1) . Our results clearly indicated that students performed better by FC method of teaching.

**Figure 1:**
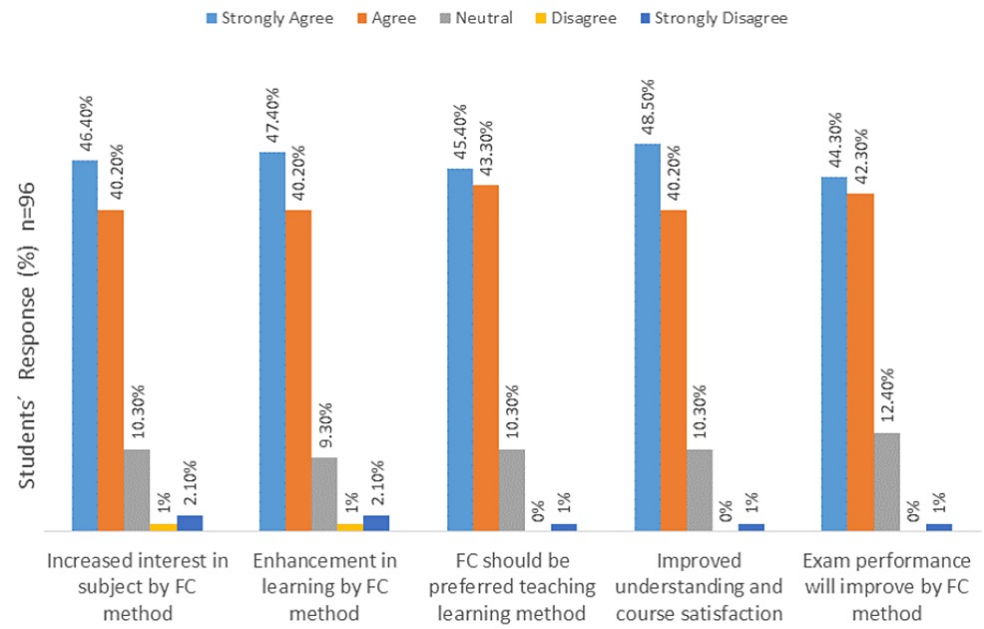
Students' perceptions toward the flipped classroom method of learning.

**Figure 2:**
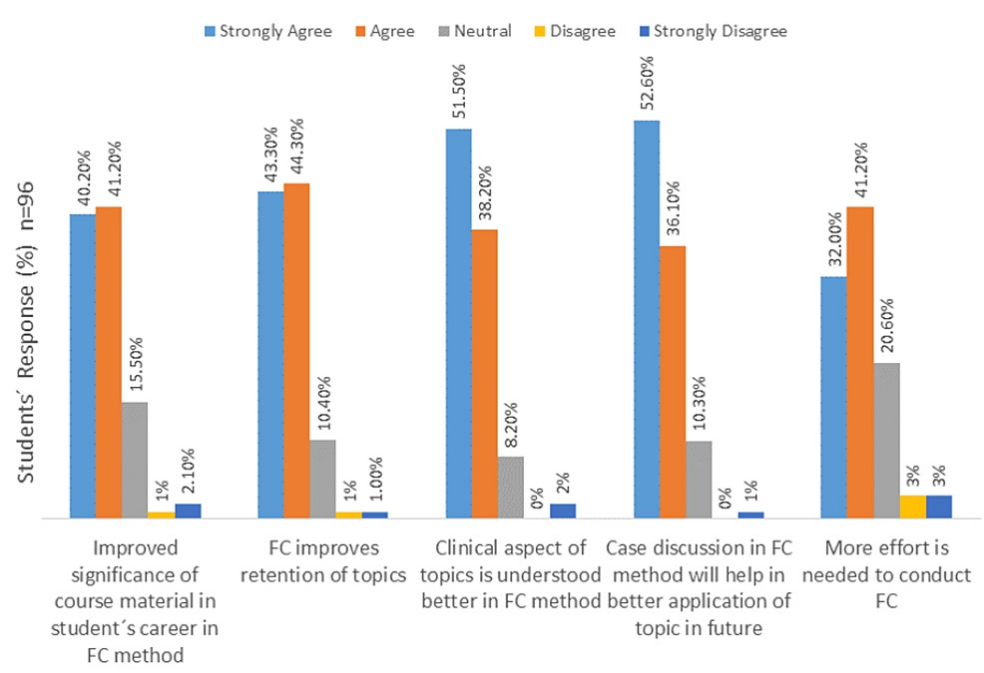
Benefits acquired by students via the flipped classroom method.

The results of students’ perceptions toward the FC method are shown in Figure 1, while the benefits expected from the FC method of teaching and learning are depicted in Figure 2.

## Discussion

The FC method is an innovative teaching-learning method where course material is provided to students before class. Students are well acquainted with the topic before the class. During teaching sessions, the baseline knowledge about the topic is utilized interactively. This is a learner-centric method that inculcates better aptitude, clinical understanding, and retention of the subject.

In the present study, the FC group performed better in post-tests as compared to the DL group for both topics. The difference in mean scores of both groups was statistically significant. Perception of students toward various aspects of the learning method was very positive, with the majority responding that it provides better understanding, increased correlation, and effective memory retention.

In an interventional study conducted by Angadi et al. on the effectiveness of FC as a teaching-learning method, the authors found that significant differences were present between pre- and post-test scores between conventional and FC groups. Further, 82% of students agreed that FC was more engaging and interesting as compared to the traditional teaching method. Overall, it was inferred that the FC method improved performance and learning experience effectively [13].

A study by Aristotle et al. for histology lectures on first-phase undergraduate students in a medical college showed significant improvement in post-test scores for the FC group (*P *< 0.0001). Students' perceptions indicated that the FC methodology for teaching histology produced excellent outcomes, with very positive responses from students [14].

In another study on 66 students, at La Guardia Community College, exam performance among students in FC increased significantly relative to the control group (mean ± SD 76.93 ± 18.33 versus 67.8 ± 18.81), with *P *< 0.001. Students felt that FC helped them learn better and connect materials to the goal of their future cause [15].

A study conducted by Verramani et al. on 130 first-year undergraduate students teaching neuroanatomy showed the mean pre- and post-test scores were 3.35 ± 1.5 and 8.12 ± 1.6, respectively. Paired t-test showed a highly significant difference between pre- and post-test scores (*P *< 0.05). The mean and SD of summative neuroanatomy scores for students who were exposed to FC methodology compared to an earlier batch of students exposed to only didactic learning were 89% ± 12% and 70% ± 14%, respectively. When the perception of students was studied, 86% of students felt FC was better for fulfilling learning objectives, 92% opined it made a clear understanding, and 78% felt that this method actively engages with the subject before class. About 93% felt that more such classes should be organized in the future [16].

Arya et al. in their study on third-year undergraduate students found that the FC model brought much better scores as compared to the traditional lecture method for training in the management of epilepsy. They concluded that FC can be used as an effective method for training primary care physicians [17].

A study conducted on 112 first-year nursing students at the College of Nursing, Sultan Qaboos University, Oman, showed that students scored significantly better on the post-test. Around 68% to 78% agreed that FC had a positive effect on improving their learning and their interest in the course content. Further, they inferred that FC was more satisfying, interesting, and capable of improving grades and learning [18].

Qualitative studies by Chowdhury et al. and Joseph et al. showed a positive perspective toward the FC method where it was preferred by 81.3% and 70% of subjects, respectively. In another study by Blair et al. on 43 postgraduate trainees, FC was the preferred mode of teaching as it helped them increase their knowledge [18-20].

The limitation of this method is that it is resource-intensive and requires time and meticulous planning for implementation. It can initially be introduced for a few clinically relevant topics and used in a hybrid manner with lectures. Additionally, the study's limitation is its small sample size.

## Conclusions

This study shows that FC is advantageous for students. It improves performance and strengthens cognition. The perception toward FC was largely positive. It improves learners' engagement, critical thinking, and clinical orientation.

This teaching-learning method requires constant motivation and consistent effort from both teachers and students to be effectively executed. Hence, it should be introduced in a graded manner, commencing with a few topics and then gradually including the majority of the syllabus. 

## References

[1] Pujol S, Baldwin M, Nassiri J (2016). Using 3 D modelling techniques to enhance teaching of difficult anatomical concepts. Acad Radiol.

[2] Singh K, Bharatha A, Sa B, Adams OP, Majumder MA (2019). Teaching anatomy using an active and engaging learning strategy. BMC Med Educ.

[3] Medical Council of India (2018). Competency Based Undergraduate Curriculum for the Indian Medical Graduate, NMC. Indian Medical Graduate.NMC. Medical Council of India, Delhi 2018.1: 1-4.

[4] Luscombe C, Montgomery J (2016). Exploring medical student learning in the large group teaching environment: examining current practice to inform curricular development. BMC Med Educ.

[5] Estes M, Ingram R, Liu J (2014). A review of flipped classroom research, practice, and technologies. Int HETL Rev.

[6] Enfield J (2013). Looking at the impact of the flipped classroom model of instruction on undergraduate multimedia students at CSUN. Tech Trends.

[7] Chen F, Lui AM, Martinelli SM (2017). A systematic review of the effectiveness of flipped classrooms in medical education. Med Educ.

[8] Lin Y, Zhu Y, Chen C (2017). Facing the challenges in ophthalmology clerkship teaching: Is flipped classroom the answer?. PLoS One.

[9] Chen KS, Monrouxe L, Lu YH, Jenq CC, Chang YJ, Chang YC, Chai PY (2018). Academic outcomes of flipped classroom learning: a meta-analysis. Med Educ.

[10] Ramnanan CJ, Pound LD (2017). Advances in medical education and practice: student perceptions of the flipped classroom. Adv Med Educ Pract.

[11] Sajid MR, Laheji AF, Abothenain F, Salam Y, AlJayar D, Obeidat A (2016). Can blended learning and the flipped classroom improve student learning and satisfaction in Saudi Arabia?. Int J Med Educ.

[12] Ain NU, Murad T, Rehman MA (2021). Flipped classroom and insight of students: progress in medical education and practices. J Res Med Dent Sci.

[13] Angadi NB, Kavi A, Shetty K, Hashilkar NK (2019). Effectiveness of flipped classroom as a teaching-learning method among undergraduate medical students - an interventional study. J Educ Health Promot.

[14] Aristotle S, Subramanian S, Jayakumar S (2021). Effectiveness of flipped classroom model in teaching histology for first-year MBBS students based on competency-based blended learning: An interventional study. J Educ Health Promot.

[15] Entezari M, Javdan M (2016). Active learning and flipped classroom hand in hand approach to improve students learning in human anatomy and physiology. Int J Higher Educ.

[16] Veeramani R, Madhugiri VS, Chand P (2015). Perception of MBBS students to "flipped class room" approach in neuroanatomy module. Anat Cell Biol.

[17] Arya V, Gehlawat VK, Rana R, Kaushik J (2020). Flipped classroom versus traditional lecture in training undergraduates in pediatric epilepsy. J Family Med Prim Care.

[18] Joseph MA, Roach EJ, Natarajan J, Karkada S, Cayaban AR (2021). Flipped classroom improves Omani nursing students performance and satisfaction in anatomy and physiology. BMC Nurs.

[19] Chowdhury TA, Khan H, Druce MR (2019). Flipped learning: turning medical education upside down. Future Healthc J.

[20] Blair RA, Caton JB, Hamnvik OR (2020). A flipped classroom in graduate medical education. Clin Teach.

